# A Comparative Study of Serum Level of Vascular Cell Adhesion Molecule-1 (sVCAM-1), Intercellular Adhesion Molecule-1(ICAM-1) and High Sensitive C - reactive protein (hs-CRP) in Normal and Pre-eclamptic Pregnancies

**Published:** 2013-05

**Authors:** Mehdi Farzadnia, Hossein Ayatollahi, Maliheh Hasan-zade, Hamid Reza Rahimi

**Affiliations:** 1 Department of Pathology, Faculty of Medicine, Mashhad University of Medical Sciences, Mashhad, Iran; 2 Departments of Hematopathology and Blood Banking; Faculty of Medicine, Mashhad University of Medical Sciences. Mashhad, Iran; 3 Cancer Molecular Pathology Research Centre, Mashhad University of Medical Sciences. Mashhad, Iran; 4 Department of Obstetrics and Gynecology, Faculty of Medicine, Mashhad University of Medical Sciences. Mashhad, Iran; 5 Student Research Committee, Department of Modern Sciences & Technologies, Faculty of Medicine, Mashhad University of Medical Sciences. Mashhad, Iran

**Keywords:** Adhesion Molecule-1 (sVCAM-1), High sensitive C-reactive protein (hs-CRP), Intercellular adhesion molecule-1(ICAM-1), Pre-eclampsia, Soluble vascular cell

## Abstract

***Objective(s):*** Pre-eclampsia is characterized by hypertension, dyslipidemia, and increased systemic inflammatory response and has been associated with an increased maternal risk of cardiovascular disease later in life. Endothelial dysfunction is thought to be a central pathogenic feature in pre-eclampsia on the basis of elevated adhesion molecules. The aim of this study was to determine the level of plasma serum level of vascular cell adhesion molecule-1 (sVCAM-1), intercellular adhesion molecule-1(ICAM-1), high sensitive C- reactive protein (hs-CRP) in pre-eclampsia and to compare hs-CRP levels between normal pregnant women, mild and severe pre-eclampsia.

***Materials and Methods*** **:** A cross-sectional study was conducted to determine the plasma concentrations of sVCAM-1, ICAM-1 and hs-CRP in peripheral blood obtained from normal pregnant women (n=40), mild pre-eclampsia (n=37) and severe pre-eclampsia (n=38). Concentrations of soluble adhesion molecule was determined with enzyme linked immunosorbent assay (ELISA).

***Results:*** There were significant difference in the means serum hs-CRP between normal pregnant women and mild pre-eclamptic women (*P*<0.05). Serum concentration of hs-CRP, sVCAM-1(ng.ml) and sICAM-1(ng.ml) were significantly higher in severe pre-eclampsia (*P*<0.05) than normal pregnancy. There were also significant differences in hs-CRP, s ICAM- 1 and in sVCAM- 1 levels between mild and severe pre-eclampsia (*P*<0.05). There was no difference in the mean plasma log sVCAM-1, sICAM-1 between normal pregnant women and mild pre-eclamptic women.

***Conclusion:*** We have determined the serum concentration of soluble adhesion molecule ICAM-1, VCAM-1 and hsCRP in normal pregnancy and pre-eclampsia. Adhesion molecule is elevated in severe pre-eclampsia compared with normal pregnancy, hsCRP are elevated in severe preeclampsia compared with mild preeclampsia and normal pregnancy and may be useful in predicting the severity of pre-eclampsia.

## Introduction

Several studies have suggested that concentrations of different soluble adhesion molecules may be useful markers of inflammation, and their concentrations have been found to be altered in conditions such as sepsis, acute coronary artery disease, renal allograft rejection, acute pancreatitis and rheumatoid arthritis (1).

Endothelial cell dysfunction is considered central to the pathophysiology of pre-eclampsia (2, 3) yet the mechanisms responsible for the development of endothelial dysfunction in this syndrome remain to be determined. Recent studies suggest that ‘normal pregnancy is associated with changes in peripheral blood leukocytes similar to those observed in sepsis (4).

Pre-eclampsia (PE) develops in 4–5% of human pregnancies. It is characterized by an elevated blood pressure and proteinuria and develops after 20 weeks of gestational age. PE can result in eclampsia when convulsions develop or manifest as the hemolysis, elevated liver enzymes and low platelet count (HELLP) syndrome. Several etiologies have been implicated in the development of pre-eclampsia. Some of them include abnormal trophoblast invasion of uterine blood vessels, immunological intolerance between fetoplacental and maternal tissues, maladaptation to the cardiovascular changes or dietary deficiencies and genetic abnormalities (5).

Adhesion molecules play a central role in the endothelial cells- leukocytes adherence and the subsequent migration of white blood cells into perivascular tissue. 

Cellular forms of adhesion molecules mediate specific steps of leukocyte–endothelial cell interaction, and have been implicated in the pathophysiology of pre-eclampsia. Soluble forms of these molecules can be detected in plasma, and their concentrations are thought to reveal the degree of activation of a particular cell type. Increase in soluble forms of vascular cell adhesion molecule 1 (sVCAM-1) and soluble forms of intercellular adhesion molecule-1 (sICAM-1) indicate endothelial cell activation/dysfunction.

C-reactive protein (CRP) is a marker of systemic inflammation(6). It has been shown that CRP is elevated in women with PE (7).

High-sensitive (hs) CRP is a protein measured by either antibodies that are labeled with an enzyme (ELISA) or a fluorescent compound or antibody-coated polystyrene beads. Determination of hsCRP has been suggested to be more sensitive than conventional measurement of CRP and provides better sensitivity in confirmation of inflammation (8).

Determination of hsCRP has been suggested to be more sensitive than conventional measurement of CRP and provides better sensitivity in confirmation of inflammation (8, 9). 

Therefore, the objective of this study was to determine whether normal pregnancy and pre-eclampsia were associated with changes in the concentrations of sICAM-1, sVCAM-1, and hsCRP.

## Materials and Methods

A cross-sectional study was designed to compare the plasma concentration of vascular cell adhesion molecule 1, intercellular adhesion molecule 1 and High-sensitive CRP in peripheral blood obtained from normal pregnant women and pregnant patients with pre-eclampsia at the Departments of Obstetrics and Gynecology of the Ghaem Academic Hospital in Mashhad University of Medical Sciences, Mashhad, Iran.

Pre-eclampsia was defined as hypertension (systolic blood pressure ≥140 mmHg and diastolic blood pressure ≥90 mmHg after 20 weeks’ gestation) and proteinuria (≥300 mg in a 24 hr urine collection or one dipstick measurement ≥1+) according to the Committee of Terminology of ACOG definition (10).

Severe preeclampsia was diagnosed on the basis of diastolic blood pressure ≥110 mmHg or significant proteinuria (dipstick measurement of ≥2+), or the presence of severity evidences such as headache, visual disturbances, upper abdominal pain, oliguria, convulsion, elevated serum creatinine, thrombocytopenia, marked liver enzyme elevation, and pulmonary edema. Normal pregnant women had no hypertension, proteinuria, and edema. The population consisted of 40 women with normal pregnancy, 37 women with mild preeclampsia, and 38 women with severe pre-eclampsia. Three groups were similar in age and body weight (mild pre-eclampsia group average age 27.4±6.4 years, severe preeclampsia 26.1±5.8 and pregnant control group 24.6± 4.2 years).

Venipuncture was performed, and the blood was collected into tubes containing ethylenediamine-tetraacetic acid (EDTA). 

The patient’s serum samples stored at -20°C until assay. The concentrations of soluble adhesion molecules were measured using enzyme-linked immunoassays (Bender Med system, Human sVCAM-1- BM232, Austria), (Bender Med System, Human sICAM-1-BM 201, Austria). For all patients and normal pregnant women, serum hsCRP level was measured with immunoturbidimetric assay (Diagnostica kit, Germany).

The sensitivity of the assay for sICAM-1 was 2.17 ng/ml for sVCAM-1 was 0.63 ng/ml. The inter-and intra-assay coefficients of variation were 7.66% and 4.1% respectively for sICAM-1 and the inter-and intra-assay coefficients of variation were 5.6 % and 3.5 %, respectively for sVCAM-1.

The lowest limit of detection was 0.1 mg/l. The maximum inter-and intra-assay coefficients of variation for the range of concentrations evaluated were 3.5% for hsCRP.

***Statistics***

The t student-test was used for comparison of proportions. A level of *P*< 0.05 was regarded as statistically significant.

## Results

This study included 40 normal pregnant women and 75 pregnant women with pre-eclampsia (37 Mild Preeclampsia and 38 Severe Pre-eclampsia). Table 1 lists the clinical characteristics of the three study groups.

**Table 1:** Clinical characteristics of the study population. Data are presented as mean±standard deviation (SD

	Normal pregnant (n=40)	Mild preeclampsia (n=37)	P	Severe preeclampsia (n=38)	P_a_	P_b_
Age(Year) mean±SD		24.6±4.2	27.4±6.4	NS	26.1±5.8	NS	NS
Gestational age at blood sampling mean±SD		37.1±2	35.7±4	NS	32.7±5.6	0.02^*^	<0.0001^*^
Body weight		71.4±10.4	77±12.5	NS	71.1±11.4	NS	NS
Birth weight (Kg) mean±SD		2.6±0.7	2.3±0.68	NS	2.1±0.97	NS	<0.05^*^
Blood pressure							
Systolic		111±14	149.1±15	<0.001^*^	154.7±19.7	NS	<0.001^*^
Diastolic		63±12	92±12	<0.001^*^	107.6±14.8	<0.001^*^	<0.001^*^
sVCAM-1(ng/ml)		971.3±253	1019±288	NS	1240±553	<0.05^*^	<0.05^*^
sICAM-1(ng/ml)		445±136	481±148	NS	606±271.8	<0.05^*^	<0.05^*^
hsCRP(mg/l)		6.7±2.0	9.2±7.1	<0.05^*^	12.8±7.3	<0.05^*^	<0.05^*^

P, comparison between normal pregnant and mild Pre-eclampsia;

P_a_, comparison between women with mild & severe pre-eclampsia;

P_b_, comparison between normal pregnant and severe pre-eclampsia;

*, statistically significant, *P* < 0.05; NS, non-significant

**Table 2:** Shows the laboratory characteristics of patients with normal pregnancy and pre-eclampsia. There was no statistically significant difference in BUN, bilirubin , creatinine, blood glucose ,uric acid, Hb, hematocrit, and platelet between mild and severe pre-eclampsia, while AST,ALT, and urine protein were significantly different between two groups (*P*<0.05)

Group	Normal Pregnancy (n=40)	Mild pre-eclampsia (n=37)	Severe pre-eclampsia (n=38)	P	P_a_	P_b_
Test						
BUN(mg/dl)	23.5±11.5	25.0±14	25.7±12	NS	NS	NS
BIL(mg/dl)						
Total	0.59±0.30	0.7±0.20	0.87±0.45	NS	NS	NS
Direct	0.19±0.12	0.26±0.11	0.31±0.17	NS	NS	NS
Creatinine(mg/dl)	0.6±0.17	0.62±0.18	0.72±0.24	NS	NS	NS
Blood glucose(mg/dl)	84.2±16.1	83.2±15.5	88.0±20.3	NS	NS	NS
Uric acid(mg/dl)	4.2±1.21	5.81±1.37	6.29±1.54	NS	NS	NS
Hb(g/dl)	12.1±1.42	11.92±1.39	12.47±1.61	NS	NS	NS
Hematocrit (%)	38.1±3.89	37.00±3.93	38.16±5.31	NS	NS	NS
Platelets(cell/μL(mm^3^)	210150±85135	208217±95180	191120±142383	NS	NS	NS
AST(U/l)	12.35±8.37	22.26±10.63	36.92±28.57	<0.05^*^	<0.05^*^	<0.05^*^
ALT(U/l)	10.48±6.01	18.39±7.09	31.24±25.28	<0.05^*^	<0.05^*^	<0.05^*^
Urine protein(g/l)	0.81±0.86	1.10±1.96	2.26±2.60	<0.05^*^	<0.05^*^	<0.05^*^

P, comparison between normal pregnant and mild Pre-eclampsia;

P_a_, comparison between women with mild &severe pre-eclampsia;

P_b_, comparison between normal pregnant and severe pre-eclampsia;

*, statistically significant, *P *< 0.05; NS, non-significant

Soluble vascular cell adhesion molecule-1 was detected in all specimens. There was no difference in the mean sVCAM-1between normal pregnant women (971.3±253) and mild pre-eclamptic women (1019±288). Patients with severe pre-eclampsia had a significantly higher mean plasma level (1240±553) than normal pregnant and mild pre-eclamptic women (*P*<0.05). Serum levels of sICAM-1 was not different statistically between the mild pre-eclamptic pregnancies (481±148 ng/ml) and normal pregnancies (445±136 ng/ml), but the concentration was higher in severe preeclampsia (606±271.8 ng/ml) compared with normal pregnancy and mild pre-eclamptic women (*P*<0.05).

In addition, hsCRP was detected in all specimens. There was a significant difference in the mean hsCRP between normal pregnant women and mild pre-eclamptic women (6.7±2 mg/l vs. 9.2±7.1 mg/l, *P*<0.05). Patients with severe preeclampsia had a significantly higher means plasma levels (12.8±7.3 mg/l) than normal pregnant (6.7±2 mg/l) and mild pre-eclamptic women (9.2±7.1 mg/l) (*P*<0.05).

## Discussion

Pre-eclampsia is characterized by hypertension, dyslipidemia, and increased systemic inflammatory response and has been associated with an increased maternal risk of cardiovascular disease later in life (11).

In recent years, endothelial dysfunction has emerged as the leading phenomenon responsible for the clinical signs of the disorder (12, 13).

Pathogenesis, pre-eclampsia is thought to be resulted from generalized endothelial dysfunction (14) Recently, increased levels of cell adhesion molecules are believed to be indicators of endothelial dysfunction in pre-eclampsia (15).

Observational and experimental studies have demonstrated an association between inflammation and endothelial dysfunction (16, 17).

Previous studies of soluble adhesion molecules in the plasma of pre-eclamptic patients yielded conflicting results (18). Some studies reported an increase in sP-selectin, sE-selectin and sICAM-1,(19-21) while others reported no changes (22). In contrast, all of the studies have reported an increase in sVCAM-1 (19, 22-25).

Two studies reported an increased plasma concentration of sPECAM-1 in pre-eclampsia.(21, 26)

(Lyall *et al) *     (24) have reported that serum levels of VCAM-1 and E-selectin were not significantly different between normal and pre-eclamptic pregnancies. Chaiworapongsa *et al* (27) suggested that serum levels of ICAM-1 were no differences between normal and pre-eclamptic pregnancies.

Our findings indicate that severe pre-eclampsia, but not mild pre-eclampsia and normal pregnancy, was associated with an increase in sVCAM-1 and sICAM-1. Similar findings have been reported by other investigators (19, 21, 24, 28).

This observation is of considerable importance, because sVCAM-1 has a distinctive pattern of regulation and is rapidly induced by pro-atherosclerotic conditions (29). We interpret the elevation in sVCAM-1 in pre-eclampsia as evidence of endothelial cell activation/dysfunction and may be useful in predicting the severity of pre-eclampsia.

In one study, plasma sICAM-1 and sVCAM-1 were analyzed between weeks 22 and 29 of gestation in 1543 pregnant women and related to the outcome of pregnancy in a prospective longitudinal study. Plasma sICAM-1 and sVCAM-1 in uncomplicated pregnancies were normally distributed and varied over a small range. In contrast, out of 177 pregnancies with complications (with a prevalence of 11.5%), 97 (55%) had sICAM-1 or sVCAM-1 concentrations above the same cutoffs weeks before the onset of disease. Therefore, mid-gestation measurements of circulating sICAM-1 and sVCAM-1 have a high predictive value and may recognize up to 55% of pregnant women who will later develop a severe pregnancy-related complication (30).

Early enhanced activation of endothelial cells, platelets and leukocytes seem to be present in pre-eclamptic patients, especially in those who develop severe pre-eclampsia (31).

There is increasing evidence that pre-eclampsia is a systemic inflammatory disease (32). CRP is responsible for the clearance of membranes and nuclear (4, 7, 32) antigens and acts as a scavenger (33).

Some reports have shown that elevated CRP levels during first trimester of pregnancy are indicative of pre-eclampsia.(34) But another study reported that serum levels of CRP at 23-25 weeks of gestation were similar in pregnant women who subsequently developed pre-eclampsia and in women without complications of pregnancy (35).

Although normal pregnancy is associated with increased pro-inflammatory markers, it has been suggested the cause of serum hsCRP elevation in the pre-eclamptic women may be a result of reduced plasma volume in these patients (32, 33).

The relationship of CRP levels and preeclampsia has already been studied and higher concentration of CRP has been reported during preeclampsia (7, 36). It has also been shown that women with a history of pre-eclampsia had increased CRP levels (37).

In our study, levels of hsCRP were found to be significantly higher in women with mild and severe pre-eclampsia than in normotensive women with similar chronological age.

(Belo *et al*) found significantly higher levels of CRP in preeclampsia, but statistical significance were lost after adjustment for maternal weight (38). Üstün *et al* showed that level of CRP to be significantly higher in women with mild and severe pre-eclampsia than in normal pregnant women with similar chronological age, gestational age, and body mass index (39).

Although inflammation may not be the exact cause of pre-eclampsia, it may enhance the pathology of the disorder in the presence of the anti-angiogenic factors (12).

Hwang HS *et al* showed that hsCRP levels were positively correlated to pregnancy duration in healthy women and could be used as a severity marker in women with severe PE (9). 

In 2011 (Can M, *et al*) found that severe pre-eclampsia group hsCRP levels were significantly higher than mild pre-eclamptic and normotensive groups (40).

There are also few studies concerning CRP levels due to severity of pre-eclampsia (41). In these studies, it has been shown that CRP levels were positively related to the degree of blood pressure elevation. In our study, we found significantly higher levels of hsCRP in severe pre-eclampsia than mild pre-eclampsia.

## Conclusion

We have determined the serum concentration of soluble adhesion molecule ICAM-1, VCAM-1 and hsCRP in normal pregnancy and pre-eclampsia. Adhesion molecules are elevated in severe pre-eclampsia compared with normal pregnancy, hsCRP is elevated in severe pre-eclampsia compared with mild pre-eclampsia and normal pregnancy and may be useful in predicting the severity of pre-eclampsia. The clinical validity of the monitoring of hsCRP needs to be established in further longitudinal studies.

## References

[1] Gearing AJ, Newman W (1993). Circulating adhesion molecules in disease. Immunol Today.

[2] Roberts JM, Taylor RN, Musci TJ, Rodgers GM, Hubel CA, McLaughlin MK (1989). Preeclampsia: an endothelial cell disorder. Am J Obstet Gynecol.

[3] Dekker GA, Sibai BM (1998). Etiology and pathogenesis of preeclampsia: current concepts. Am J Obstet Gynecol.

[4] Sacks GP, Studena K, Sargent K, Redman CW (1998). Normal pregnancy and preeclampsia both produce inflammatory changes in peripheral blood leukocytes akin to those of sepsis. Am J Obstet Gynecol.

[5] Sibai BM (2003). Diagnosis and management of gestational hypertension and preeclampsia. Obstet Gynecol.

[6] Ridker PM, Glynn RJ, Hennekens CH (1998). C-reactive protein adds to the predictive value of total and HDL cholesterol in determining risk of first myocardial infarction. Circulation.

[7] Teran E, Escudero C, Moya W, Flores M, Vallance P, Lopez-Jaramillo P (2001). Elevated C-reactive protein and pro-inflammatory cytokines in Andean women with pre-eclampsia. Int J Gynaecol Obstet.

[8] Yu H, Rifai N (2000). High-sensitivity C-reactive protein and atherosclerosis: from theory to therapy. Clin Biochem.

[9] Hwang HS, Kwon JY, Kim MA, Park YW, Kim YH (2007). Maternal serum highly sensitive C-reactive protein in normal pregnancy and pre-eclampsia. Int J Gynaecol Obstet.

[10] Cunningham FG, Williams JW, Leveno KJ, Bloom S, Hauth JC, Rouse DJ (2009). Williams obstetrics.

[11] Freeman DJ, McManus F, Brown EA, Cherry L, Norrie J, Ramsay JE (2004). Short- and long-term changes in plasma inflammatory markers associated with preeclampsia. Hypertension.

[12] Ramma W, Ahmed A (2011). Is inflammation the cause of pre-eclampsia?. Biochem Soc Trans.

[13] Ahmed A (2011). New insights into the etiology of preeclampsia: identification of key elusive factors for the vascular complications. Thromb Res.

[14] Friedman SA, Taylor RN, Roberts JM (1991). Pathophysiology of preeclampsia. Clin Perinatol.

[15] Roberts JM (1998). Endothelial dysfunction in preeclampsia. Semin Reprod Endocrinol.

[16] Mantovani A, Dejana E (1989). Cytokines as communication signals between leukocytes and endothelial cells. Immunol Today.

[17] Greer IA, Dawes J, Johnston TA, Calder AA (1991). Neutrophil activation is confined to the maternal circulation in pregnancy-induced hypertension. Obstet Gynecol.

[18] Molvarec A, Szarka A, Walentin S, Beko G, Karadi I, Prohaszka Z (2011). Serum leptin levels in relation to circulating cytokines, chemokines, adhesion molecules and angiogenic factors in normal pregnancy and preeclampsia. Reprod Biol Endocrinol.

[19] Higgins JR, Papayianni A, Brady HR, Darling MR, Walshe JJ (1998). Circulating vascular cell adhesion molecule-1 in pre-eclampsia, gestational hypertension, and normal pregnancy: evidence of selective dysregulation of vascular cell adhesion molecule-1 homeostasis in pre-eclampsia. Am J Obstet Gynecol.

[20] Austgulen R, Lien E, Vince G, Redman CW (1997). Increased maternal plasma levels of soluble adhesion molecules (ICAM-1, VCAM-1, E-selectin) in preeclampsia. Eur J Obstet Gynecol Reprod Biol.

[21] Krauss T, Osmers R, Beran J, Diedrich F, Fleckenstein G, Kuhn W (1998). [Soluble adhesion molecules in patients with pre-eclampsia]. Zentralbl Gynakol.

[22] Daniel Y, Kupferminc M, Baram A, Jaffa A, Wolman I, Shenhav M (1998). Plasma soluble endothelial selectin is elevated in women with pre-eclampsia. Hum Reprod.

[23] Krauss T, Azab H, Dietrich M, Augustin HG (1998). Fetal plasma levels of circulating endothelial cell adhesion molecules in normal and preeclamptic pregnancies. Eur J Obstet Gynecol Reprod Biol.

[24] Lyall F, Greer IA, Boswell F, Macara LM, Walker JJ (1994). The cell adhesion molecule, VCAM‐1, is selectively elevated in serum in pre‐eclampsia: does this indicate the mechanism of leucocyte activation?. Br J Obstet Gynaecol.

[25] Farzadnia M, Ayatollahi H, Maliheh HM, Rahimi HR (2011). A comparative study of serum soluble vascular cell adhesion molecule-1(svcam-1) and intercellular adhesion molecule-1(icam-1) in normal pregnant adhesion molecule-1(icam-1) in normal pregnancy. Clin Biochem.

[26] Zeisler H, Livingston JC, Schatten C, Tempfer C, Knofler M, Husslein P (2001). Serum levels of adhesion molecules in women with pregnancy-induced hypertension. Wien Klin Wochenschr.

[27] Chaiworapongsa T, Romero R, Yoshimatsu J, Espinoza J, Kim YM, Park K (2002). Soluble adhesion molecule profile in normal pregnancy and pre-eclampsia. J Matern Fetal Neonatal Med.

[28] Madazli R, Budak E, Calay Z, Aksu MF (2000). Correlation between placental bed biopsy findings, vascular cell adhesion molecule and fibronectin levels in pre-eclampsia. BJOG.

[29] Ley K, Huo Y (2001). VCAM-1 is critical in atherosclerosis. J Clin Invest.

[30] Krauss T, Emons G, Kuhn W, Augustin HG (2002). Predictive value of routine circulating soluble endothelial cell adhesion molecule measurements during pregnancy. Clin Chem.

[31] Chavarría ME, Lara-González L, García-Paleta Y, Vital-Reyes VS, Reyes A (2008). Adhesion molecules changes at 20 gestation weeks in pregnancies complicated by preeclampsia. Eur J Obstet Gynecol Reprod Biol.

[32] Redman CW, Sacks GP, Sargent IL (1999). Preeclampsia: an excessive maternal inflammatory response to pregnancy. Am J Obstet Gynecol.

[33] Du Clos TW (1996). The interaction of C-reactive protein and serum amyloid P component with nuclear antigens. Mol Biol Rep.

[34] Tjoa ML, van Vugt JM, Go AT, Blankenstein MA, Oudejans CB, van Wijk IJ (2003). Elevated C-reactive protein levels during first trimester of pregnancy are indicative of preeclampsia and intrauterine growth restriction. J Reprod Immunol.

[35] Savvidou MD, Lees CC, Parra M, Hingorani AD, Nicolaides KH (2002). Levels of C-reactive protein in pregnant women who subsequently develop pre-eclampsia. BJOG.

